# Steps for Preventing Infectious Diseases in Women^1^

**DOI:** 10.3201/eid1011.040555

**Published:** 2004-11

**Authors:** Mirta Roses Periago, Ricardo Fescina, Pilar Ramón-Pardo

**Affiliations:** * Pan American Health Organization, Regional Office of the World Health Organization, Washington, DC, USA

**Keywords:** Infectious diseases, women’s health, public health, poverty, race and ethnicity, gender, conference report

## Abstract

Communicable diseases account for approximately 25% of deaths in most Latin American and Caribbean countries; illness from communicable diseases reaches 40% in developing countries. Mainly affected are poor women in rural areas. A medical approach is not sufficient to implement effective infectious disease prevention strategies in women, which would offset these numbers. Health policies must be changed, and social restrictions that circumscribe women need to be eliminated. In the long run, the only solution is to improve women's socioeconomic status. The following three steps are necessary for developing a prevention strategy: 1) a gender perspective must be incorporated into infectious disease analysis and research to target policies and programs. Data collected must be disaggregated by sex, age, socioeconomic status, education, ethnicity, and geographic location; 2) models must be developed and implemented that address gender inequities in infectious diseases in an integrated manner; and 3) outreach activities must be supported, using information, education, and communication strategies and materials for advocacy and training. Active participation of civil society groups is key to translating the strategy into specific interventions.

## Abstract 2

Infectious disease prevention must take into account women’s unique vulnerabilities and must consider biomedical, social, economic, and personal factors.

"…as the Millennium Declaration made clear, gender equality is not only a goal in its own right; it is critical to our ability to reach all the others."

—Kofi Annan, Secretary-General of the United Nations

In most Latin American and Caribbean countries, communicable diseases cause approximately 25% of deaths. This overall rate varies from country to country; it is higher in less developed countries. Communicable diseases; perinatal conditions and complications of pregnancy, childbirth, and postpartum; and nutritional diseases represent approximately 5% of the illness in industrialized countries. That percentage climbs to 40% in developing countries and reaches 50%–60% in some areas where HIV/AIDS epidemics are widespread (*1*). As a consequence, a large decrease in deaths of women would be expected if infectious diseases decreased through effective prevention strategies.

In the developing world, infectious diseases mainly affect women in rural areas (*2*). Poor women are at a greater disadvantage for coping with these diseases because of their social environment. Several infectious diseases can be successfully treated with available drugs, and well-known methods are available to prevent many diseases. Much could be done to improve health services, including implementing earlier case detection and better treatment regimens.

A medical approach will not succeed by itself, however. Success will only be achieved when coupled with behavioral changes and a breakdown of social barriers that restrict women. In the long run, the only solution is to improve women's socioeconomic status; this requires educating them, which, in turn, accelerates their social and economic progress.

## Women and Communicable Diseases: A Situational Analysis

An examination of health policies over the last 2 decades in most of the Americas illustrated the following points: 1) Women's health, in and of itself, rarely has been at the forefront of international development programs or national health planning and policies. 2) The focus on women's health in developing countries has been motivated largely by other concerns. As a rule, women have been viewed as the vehicles through which specific goals, such as family planning and child survival, could be achieved, rather than as the primary beneficiaries of or the partners in development programs. 3) The global agenda for preventing communicable diseases among women rests on two premises, namely, that understanding women's health in developing countries, particularly the health risks they face, is important for instituting appropriate interventions to address women's specific health needs, and that women's participation in health promotion and disease prevention is key to the health of families and communities worldwide.

Evidence collected in studies conducted in various countries shows that macrodeterminants—such as gender, ethnic origins, or race—play a major role in the degree of access to services and in the health status of populations. A study of racial inequities in health conducted in Brazil (*3*) examined infant deaths in relation to both race and level of education of the mother. For illiterate mothers, infant death rates neared 120/1,000 for black women, 110/1,000 for mulatto and dark-skinned women, and 95/1,000 for white women. Among mothers who had >8 years of education, the rates were much lower—82 for black women, 70 for mulatto and dark-skinned women, and 57 per 1,000 for white women. These rates indicate disparities according to race. Black women need 4–7 years of education before the death rates of their infants are as low as those of infants born to uneducated white women, demonstrating the strength of the effect of ethnically based discrimination in health.

In Latin America and the Caribbean, the death rate in the population aged 15 to 59 years old is higher in men than in women; in the poorest groups, however, the risk for death in men and women is nearly equal. In 13 Latin American and Caribbean countries, the death ratio between poor and not-poor populations is three times higher for men and seven times higher for women.

The higher ratio for women is partially explained because the number of deaths during the pregnancy and childbearing years is significantly higher in those at lower socioeconomic levels. This fact captures one of the greatest inequities in health, given that most childbirth-related deaths are preventable. Infections after childbirth and after abortions take a considerable toll in countries where maternal deaths are >50 per 100,000 live births.

An estimated 25 million cases of sexually transmitted infections (STI) occur each year in the Americas. An estimated 330,000 pregnant women have positive serologic test results for syphilis every year. The 3.1%^2^ maternal positive serologic rate suggests that this disease is a major contributor to illness in women and infant deaths. Moreover, the HIV infection rate in the Caribbean is rising faster among women than among men. Of all the HIV infections in the region, women account for 25% in Latin America and 35% in the Caribbean; the sex ratio in those countries where the epidemic is widespread approaches 1:1.

Communicable diseases in developing countries are largely diseases of poverty (*2*). The poor are at most risk because of their precarious living conditions, often inadequate health services, and lack of access to care. In many cultures, the lower value assigned to women translates into higher levels of suffering, with infectious diseases accounting for 33% of all causes of death among women (*4*).

A long history of gender discrimination also leads to inequalities that perpetuate a lack of access to resources and services for women and their children. Almost 70% of the 1.2 billion people worldwide living in extreme poverty are women (*5*), who experience more illness and are less likely to receive medical treatment. Women report 15% more health problems (diseases and accidents) than do men. Yet, women's use of the health services is only 2% higher than men's. Furthermore, this tendency for women to have even this modestly greater use of health services than men disappears in the lowest income quintile, where, paradoxically, the gender gap in health need is widest.

Health data from Guatemala (*6*) indicate that there is a persistent gap in access to health care between indigenous and nonindigenous groups. Indigenous groups get less prenatal care than nonindigenous groups (45% vs. 67%, respectively). For example, tetanus vaccination rates for indigenous and nonindigenous groups are 46% and 62%, respectively.

Ethnic origins, too, function as an invisible barrier that hinders access to health services. In the Municipality of São Paulo, the health system offers retrovirus treatment for HIV patients, which has decreased death rates. A closer look at the heath statistics of the municipality's department of health 2003 (*7*), however, shows that the risk for death from HIV/AIDS among black women is four times higher than that among Caucasian women.

## Gender Framework for Infectious Diseases

The World Health Organization has developed a framework (*8*) that outlines the parameters of a gender approach for understanding the differential impact of communicable diseases on women and men (Figure). Few studies have focused on the economic and productive impact of infectious diseases, considering the cost of reduced or lost productivity and expenditures on drugs and health care at the individual worker level. For the most part, these studies have failed to capture the economic impact of disease within the household. And it is precisely at the household level that women are most affected, both as caregivers and as patients.

**Figure:**
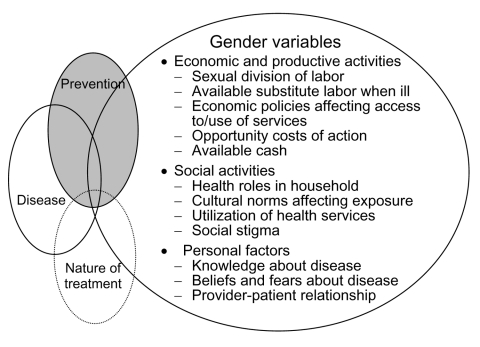
Framework on gender variables developed by the World Health Organization Special Programme for Research and Training in Tropical Diseases.

In most developing countries, unemployment is higher among women, and when women are employed, their salaries are generally much lower than men's (*9*). These conditions mean that women have fewer resources than men, yet spend more of their own income on health care for their children and other family members. Structural adjustment programs have placed additional burdens on women. Reductions in state-supported healthcare programs have resulted in reduced access to health care for the poorest populations, and long waits in clinics have serious repercussions for women's time for other activities.

The framework also includes many social determinants of infectious diseases, such as domestic and social roles and responsibilities, cultural norms affecting exposure, available support networks, social stigmas, use and quality of health services, and decision-making power within the household and community. In many cultures, men are still given better care within the family, as well as outside. Women's lower status in the household affects their access to information about health and preventive measures, as well as their ability to seek treatment. Evidence suggests that women's ability to make decisions has considerable influence on the health of their children. When health messages meant for women are directed to men, their direct influence on women's understanding and behavior may be greatly diluted.

On the other hand, when men have taken responsibility for their children's health care, results have been dramatic. In Ghana, for example, the fathers' participation in the decision to immunize their children not only increased vaccination rates but also led to earlier immunization and more timely completion of the immunization schedule (*10*). These findings show that health programs can be improved by educational messages that promote the sharing of child care and are directed to the family as a whole, not just to the mother, as has traditionally been done.

Physicians' tendency to release knowledge only when they consider it necessary has been found to curtail women's understanding and adherence to medical advice. For example, a study in rural Bolivia found that women living in areas where Chagas' disease was endemic were able to recognize triatomine bugs and had seen them in their houses, but 59% did not know that they could transmit disease (*11*). Health education programs are based on modern biomedical explanations that often are too abstract for persons to link to their own environment. In addition, health authorities do not sufficiently build upon local perceptions of disease and disease transmission.

Men and women experience disease differently in important ways. These differences can be grouped into social, economic, and personal consequences. Studies of the economic consequences of malaria in Colombia (*12*) found that women waited longer to seek treatment because their household work was essential for the functioning of other family members. They also took longer to recover than men and often returned to work while still debilitated. Men's illnesses were diagnosed and treated earlier, and men received better care while recovering. Although women or other family members took over the work of the men who became ill, much of women's work was left undone until they could return to it after recovering.

Gender differences in the social and personal consequences of infectious diseases are illustrated by studies on stigmatizing diseases such as leprosy or HIV/AIDS. Generally, women suffer greater discrimination and are more likely to be held responsible for their illness and to be isolated from their families and communities (*13*).

## Response of the Pan American Health Organization (PAHO)

### An Integrated Model To Address Gender Inequities

Since the 1990s, PAHO's Gender and Health Unit has worked at the regional, national, and community levels to advocate for, and involve communities in, formulating gender-sensitive health policies. This unit has developed an integrated model to address gender inequities. The model was initially validated by using gender-based violence, but it can be adapted to apply to communicable diseases. The model involved communities providing care and support to persons affected by gender-based violence through networks that plan, implement, and monitor several activities. First, health services are often the initial contact point, so health providers are trained to screen women for gender-based violence during routine healthcare visits. A situation analysis then assesses the prevalence of gender-based violence in the community and identifies organizations and persons who help victims. Community organizations and leaders are then mobilized into support and service networks that meet to plan activities. Finally, replications of the community networks at the regional and national levels advocate for policies, legislation, and resources.

### Millennium Development Goals

The United Nations Millennium Declaration (2000) prioritizes development and freedom for all the peoples of the world. As a way to monitor national and international progress in this regard, the United Nations and other international organizations formulated the Millennium Development Goals, which have rapidly become the primary focus of international development efforts as countries and organizations strive to meet them.

Within these goals, gender equality and women's empowerment are acknowledged as central to development through goal number 3. However, since gender is an issue that affects practically every aspect of people's lives, it has also becomes central to achieving the remaining seven goals.

### Gender-based Violence and STI

A growing number of studies have documented the high prevalence of intimate partner and sexual violence against women worldwide. This violence increases women's vulnerability to HIV and STI both directly, through forced sex, and indirectly by constraining their ability to negotiate sexual contact and the use of condoms. In addition, sexual abuse during childhood has been associated with high-risk behavior later in life, which also increases the risk for HIV infection (*14*).

PAHO is promoting primary prevention of STI/HIV/AIDS through several strategies. These include increasing access to reproductive health services; reducing violence against women; protecting women's rights and their property; ensuring women's and girls' access to health services and treatment; and supporting educational efforts to combat stigmatization and discrimination.

Infectious disease prevention must include several steps. First, a gender perspective must be incorporated into infectious disease analysis and research so that policies and programs can be targeted more effectively. An initial step should be the collection of data disaggregated not only by sex, but also by age, socioeconomic status, education level, ethnicity, and geographic location. Second, models that address gender inequities in infectious diseases in an integrated manner need to be developed and implemented. Third, support outreach activities, using IEC (information, education, and communication) strategies and materials for advocacy and training, need to be set up.

## Next Steps in Prevention

The failure to acknowledge that gender and poverty interact to place women at particular health risk contributes to stereotypes used in infectious diseases research and control (*15*). Gender stereotypes can be reflected in the delivery of health services in two ways. First, women and men have different vulnerabilities to infectious diseases that have to do with biologic, social, and cultural factors. Many times, similar prevention strategies are provided for men and women, even when their needs are not the same (e.g., HIV/AIDS prevention campaigns directed to male and female adolescents). Secondly, infectious disease prevention and control programs often reinforce gender stereotypes. For example, programs often focus exclusively on mothers to be responsible for children's health, oral rehydration therapy, or supervision of drug therapy for tuberculosis.

Gender interacts with biologic differences and social factors. It affects access to health care, health-seeking behavior, health status, and the way health policies and programs are developed and implemented (*16*). More than a variable, gender is a construct that underpins the way health sciences and the health system are organized (*17*). Enormous changes need to take place in the study and control of infectious diseases. Social and behavioral scientists have long argued that these diseases will not be eliminated without attention to social inequalities.

The key for putting gender values firmly in place within prevention strategies is a change of philosophy at all levels of the health sector, from the political to the healthcare levels. The traditional male model for prevention needs to be seen as inadequate. Rather, both gender equity and equality are required to attain health for all. The steps for developing a gender-sensitive prevention strategy include political commitment, knowledge building, and development of the technical capacity and awareness to implement and evaluate preventive strategies

### Political Commitment

Political commitment is necessary to empower women and to address gender inequalities. This commitment should translate into prevention strategies specifically targeted to women. Integrating a gender analysis into health policies and programs is necessary for achieving the Millennium Development Goals.

These goals acknowledge gender equity as an important prerequisite for development. In fact, the third Millennium Development Goal deals specifically with gender (*18*). A core strategy in working to achieve these goals is ensuring that a gender-sensitive approach is incorporated into strategies and interventions for infectious diseases (e.g., for HIV/AIDS, tuberculosis, and malaria).

Social civil organizations, such as the "Red de Salud de la Mujer de Latinoamérica y el Caribe," are important stakeholders to advocate for political commitment and to foster the implementation of norms and policies. Political commitment should also be translated into the necessary budget allocations, to be incorporated as a separate line item in programs, to allow planning, implementation, and training in a gender-sensitive approach to preventing infectious diseases.

Gender should be mainstreamed through each health organization, from the World Health Organization to governmental and nongovernmental organizations. Women's involvement in policy and program development at the highest levels will facilitate the integration of gender into health programming and policymaking.

### Knowledge Building

#### Epidemiologically Disaggregated Data

Studies demonstrate that gender analysis provides a more comprehensive understanding of the epidemiology of health problems, including that of infectious diseases. Analysis of health determinants, impact of disease, and health-seeking behavior should be disaggregated by sex to determine the different factors that affect women. Health statistics based on official data from health services may underestimate female illness and death from infectious diseases because women often do not go to these centers for detection and treatment of their health problems, as has been shown in a study on cutaneous leishmaniasis in Colombia (*19*).

#### Research

Much of the gender research in infectious diseases is outdated, limited, or inconclusive. Socioeconomic and health factors in relation to infectious diseases and gender have been established, but epidemiologic data are sparse. For example, tuberculosis is one of the most important causes of death among women, killing more women that all maternal causes of death. Sex-related research on tuberculosis is lacking, however (*20*). To address the impact of sex in infectious diseases, we need to assess the magnitude of the problem, study sex differences, and then pilot interventions to address the problems identified.

Gender also affects the research questions asked, the way data are examined, and the way male or female clients are treated when they come to the health center. Gender is more than a variable to be manipulated; it is an organizing principle of society. The mainstreaming of gender-sensitive research needs to be linked to the mainstreaming of gender sensitivity in infectious diseases programs, including prevention programs.

#### Development of Evidence-based Strategies and Interventions Addressing Women's Specific Needs

Medical and social scientists must seriously consider gender, its interaction with physiologic and immunologic factors, and the ways in which men and women can be protected from or put at risk for communicable diseases by that interaction. Interventions in communicable diseases must be planned with an understanding of the way in which gender influences the degree to which men and women, as persons and population groups, have access to and control of the resources needed to protect their own health and that of family and community members. Those involved in infectious disease prevention—be they working in vector control, vaccine and drug development, improvement of surveillance and monitoring systems, or health work in countries with endemic diseases—need to be aware that gender structures the way they assess problems.

Operational research is needed to determine how best to ensure that global strategies (e.g., the directly observed treatment short course [DOTS], the Roll Back Malaria initiative, the so-called 3 by 5 Initiative) are gender-sensitive. After research results have been analyzed, interventions should be planned, implemented, and evaluated. A gender equality approach should be promoted in all preventive activities, from an awareness campaign of malaria to an HIV voluntary counseling and testing intervention.

#### Development of Technical Capacity and Awareness To Implement and Evaluate Preventive Strategies

Linking gender to training and performance of health professionals is critical. An understanding of gender and its implications for health and health-seeking behavior should be incorporated into training of health professionals and development of health sector responses.

Promotion of girls' education and women's empowerment should be addressed through interagency collaboration. Research has shown repeatedly that health information provided to rural women in developing countries is incomplete and frequently ill-adapted to their priorities and needs. Many such women have not benefited from formal education and are unable to read. They cannot understand the writing on health education posters or directions on medications. Conducting a gender analysis of epidemiologic data improves the detection and treatment of infectious diseases in underreported groups (*21*), which, in turn, provides the cornerstone for designing adequate preventive strategies.

## Conclusion

When planning and implementing infectious disease prevention's strategies, gender is an issue that has been neglected by infectious disease programs. A gender-based approach to communicable diseases helps to elucidate the various factors involved in the impact of infectious diseases in the population. After more than a decade of work on gender approaches, a new method is needed to translate the frameworks and theories into specific public health interventions so that gender inequalities are minimized.

Partnerships with the civil society should advocate for decreasing gender inequalities in health, including infectious diseases. This advocacy can foster the political commitment to institutionalize policies in norms and protocols that would decrease these inequalities. Agencies should promote building technical capacity and gender-sensitivity awareness in both developing and industrialized countries. Incorporating a gender perspective into health policies and programs is necessary for improving the coverage and effectiveness of health programs.

## Footnotes

2 Rate for Latin America and Caribbean, 2002.
